# Supplementary material for: Conditional chemoconnectomics (cCCTomics) as a strategy for efficient and conditional targeting of chemical transmission
Source: eLife. 2024 Apr 30;12:RP91927. doi: 10.7554/eLife.91927 (PMC11060718; doi:10.7554/eLife.91927)
Supplement: Figure 3—source data 3. [file elife-91927-fig3-data3.zip › Figure 3 source data3 part 2.zip!/Figure 3-Source data 3-part2/1025-13-CRISPResso_Report_ack040l3.zip!/CRISPResso_on_1025_15/9.Alleles_frequency_table_around_sgRNA_agcacaaacttggcggtgac.pdf]

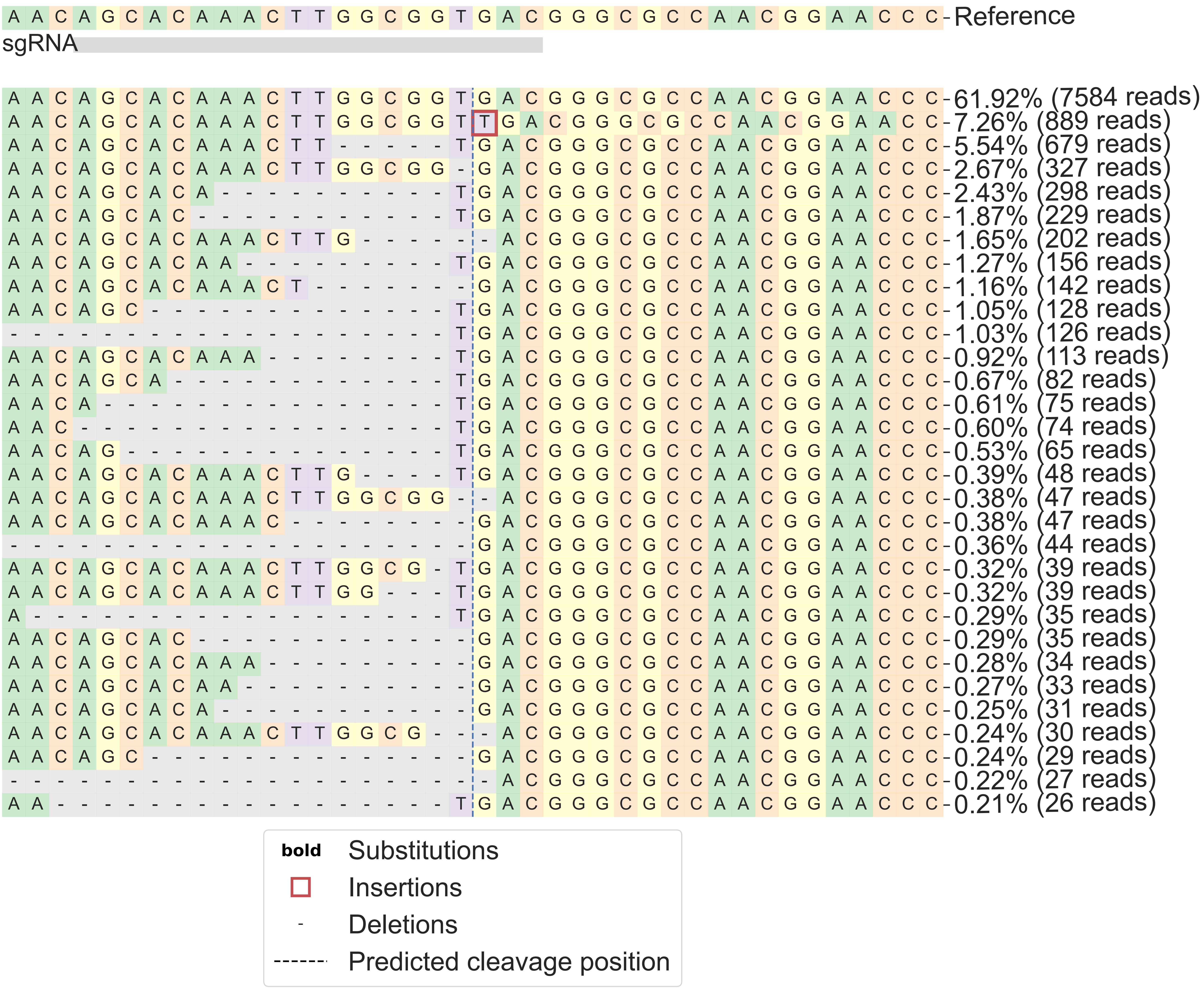

AACAGCACAAACTTGGCGGTGACGGGCGCCAACGGAACCC Reference
sgRNA
61.92% (7584 reads)
7.26% (889 reads)
5.54% (679 reads)
2.67% (327 reads)
2.43% (298 reads)
1.87% (229 reads)
1.65% (202 reads)
1.27% (156 reads)
1.16% (142 reads)
1.05% (128 reads)
1.03% (126 reads)
0.92% (113 reads)
0.67% (82 reads)
0.61% (75 reads)
0.60% (74 reads)
0.53% (65 reads)
0.39% (48 reads)
0.38% (47 reads)
0.38% (47 reads)
0.36% (44 reads)
0.32% (39 reads)
0.32% (39 reads)
0.29% (35 reads)
0.29% (35 reads)
0.28% (34 reads)
0.27% (33 reads)
0.25% (31 reads)
0.24% (30 reads)
0.24% (29 reads)
0.22% (27 reads)
0.21% (26 reads)
bold Substitutions
Insertions
Deletions
Predicted cleavage position
